# Diagnostic efficacy of ANAs Combined with serum PTX3 and CCL19 for systemic lupus erythematosus

**DOI:** 10.12669/pjms.42.6.14767

**Published:** 2026-06

**Authors:** Qing Liu, Jianqiao Zhang, Meichen Liu, Dong Chen, Meng Zhang

**Affiliations:** 1 Qing Liu, Department of Laboratory, Affiliated Hospital of Hebei University, Baoding 071000, Hebei, China; 2 Jianqiao Zhang, Department of Laboratory, Affiliated Hospital of Hebei University, Baoding 071000, Hebei, China; 3 Meichen Liu, Department of Laboratory, Affiliated Hospital of Hebei University, Baoding 071000, Hebei, China; 4 Dong Chen, Department of Laboratory, Hebei Province Sixth People’s Hospital, Baoding 071000, Hebei, China; 5 Meng Zhang, Department of Laboratory, Affiliated Hospital of Hebei University, Baoding 071000, Hebei, China

**Keywords:** Antinuclear antibody spectrum, Chemokine ligand 19, Diagnostic efficacy, Pentraxin 3, Systemic lupus erythematosus

## Abstract

**Objective:**

To investigate the diagnostic efficacy of antinuclear antibody spectrum(ANAs) combined with serum pentraxin 3(PTX3) and chemokine ligand 19(CCL19) for systemic lupus erythematosus (SLE).

**Methodology:**

This is a retrospective study. A total of 80 patients with early atypical SLE who presented to the Department of Rheumatology and Immunology of Affiliated Hospital of Hebei University between January 2022 to June 2025 were retrospectively enrolled as the experimental group. Eighty patients with other autoimmune diseases admitted during the same period were assigned to the disease control group, while 60 gender- and age-matched healthy volunteers from the hospital’s Physical Examination Center served as the healthy control group. The Western blot assay was utilized to determine the expression of ANAs in serum, whereas the enzyme-linked immunosorbent assay(ELISA) was adopted for quantifying serum PTX3 and CCL19 levels.

**Results:**

In the experimental group, complement C3, complement C4, platelet count, white blood cell count, and hemoglobin levels were significantly lower than those in both the healthy control and disease control groups, while serum creatinine and blood urea nitrogen levels were markedly elevated(all *P*< 0.05). Serum PTX3 and CCL19 levels in the experimental group were significantly higher than those in the other two groups(all *P*< 0.05). Compared with the single detection of ANAs, PTX3, or CCL19 alone, the combined detection of these three indicators exhibited significantly higher sensitivity(96.25%), accuracy(90.45%), and negative predictive value(97.60%) (all *P*< 0.05).

**Conclusion:**

ANAs, serum PTX3, and CCL19 can all act as potential diagnostic biomarkers for SLE. The combined detection of these three indicators effectively enhances the diagnostic efficiency of SLE, which is superior to single-marker detection.

## INTRODUCTION

Systemic lupus erythematosus (SLE) is a severe multisystem autoimmune disorder characterized by inflammation and immune-mediated injury to multiple organ systems, including the skin and mucous membranes, musculoskeletal system, hematological system, and renal system. It is estimated that approximately 3.4 million individuals worldwide have been diagnosed with SLE.^1^,^2^ The clinical manifestations of SLE are complex and diverse, with early symptoms often being atypical, leading to delays in diagnosis that compromise treatment timing and long-term prognosis.^3^ Therefore, the rapid and efficient diagnosis of SLE holds great clinical significance.

Antinuclear antibody (ANA) serves as the primary immunodiagnostic tool for SLE. With advances in detection technologies, the ANA spectrum has expanded to include multiple specific autoantibodies such as anti-SSA, anti-nucleosome, anti-histone, and anti-ribosomal P protein. These antibodies not only play a pivotal role in the classification and diagnosis of SLE but also reflect the immune activity of the disease to a certain extent.^4^,^5^ Pentraxin 3 (PTX3), a member of the long pentraxin family, can be rapidly secreted by monocytes, endothelial cells, and other cell types upon inflammatory stimulation, and is involved in innate immune responses and tissue repair processes. Accumulating evidence has demonstrated that PTX3 expression is upregulated in SLE patients and correlates with disease activity, indicating its potential utility in diagnosis and disease monitoring.^6^

Chemokine ligand 19 (CCL19) is a chemokine whose elevated levels may desensitize chemokine receptors on the surface of activated lymphocytes, resulting in the loss of normal defense mechanisms and subsequent massive inflammatory responses. Research has identified CCL19 as a potential biomarker for SLE disease activity^7^. Based on this, the present study was designed to explore the diagnostic efficacy of antinuclear antibody spectrum (ANAs) combined with serum PTX3 and CCL19 for SLE, assess the advantages of their combined detection, and offer novel insights and auxiliary tools for the early identification and clinical screening of SLE.

## METHODOLOGY

A total of 80 patients with early atypical SLE who presented to the Department of Rheumatology and Immunology of Affiliated Hospital of Hebei University between January 2022 and June 2025 were retrospectively enrolled as the experimental group. Eighty patients with other autoimmune diseases admitted during the same period were included as the disease control group. Sixty gender- and age-matched healthy volunteers from the hospital’s Physical Examination Center during the same period served as the healthy control group.

### Ethical approval:

The study was approved by the Institutional Ethics Committee of Affiliated Hospital of Hebei University (No.:HDFYLL-KY-2022-020; Date: December 30, 2022), and written informed consent was obtained from all participants.

### Experimental group inclusion criteria:

- Meeting the relevant diagnostic and therapeutic guidelines for SLE.^8^
- Aged ≥ 18 years.
- First visit or non-acute exacerbation, with no prior administration of glucocorticoids or immunosuppressants within the preceding two weeks.
- Complete clinical documentation and laboratory test results.

### Experimental group exclusion criteria:

- Comorbidity with other autoimmune diseases.
- Presence of active infection, malignant tumor, severe liver or kidney dysfunction, or cardiovascular diseases.
- Pregnant or lactating women.
- Incomplete clinical data.

### Disease control group inclusion criteria:

- Diagnosed with other autoimmune diseases such as rheumatoid arthritis, Sjögren’s syndrome, or systemic sclerosis.
- No evidence of SLE diagnosis.
- Age and gender matched with the experimental group.

### Disease control group exclusion criteria:

- Comorbidity with SLE or clinical suspicion of SLE
- Comorbidity with severe underlying diseases such as infection, tumor, or organic mental illness.
- Pregnant or lactating women
- Incomplete clinical data.

### Healthy control group inclusion criteria:

- No autoimmune diseases detected during physical examination.
- Age and gender matched with the experimental group.
- Routine blood tests, liver and kidney function, and immune indicators (C3/C4) within normal ranges
- Negative autoantibodies or only low-titer non-specific positive ANA (< 1:100).

### Healthy control group exclusion criteria:

- History of any chronic diseases (e.g., autoimmune diseases, tumors, infections, major organ dysfunction).
- Pregnancy or lactation
- Incomplete test data or unavailable serum samples.

Clinical data of SLE patients were collected, including general demographic data (age, gender, BMI) and clinical laboratory indicators. Routine blood tests (white blood cell count, hemoglobin, platelet count) were detected by an automatic hematology analyzer; renal function indicators (serum creatinine, blood urea nitrogen) were measured by an automatic biochemical analyzer; and complement C3 and C4 were detected by immunoturbidimetry.

*Detection of ANAs positivity rate:* ANAs were detected using an ANAs Western blot kit produced by EUROIMMUN (Hangzhou) Medical Laboratory Diagnosis Co., Ltd., with fully automated operation performed by the EUROBlotMaster II automatic Western blot analyzer (EUROIMMUN GmbH, Germany). A total of 12 specific autoantibodies were tested, including anti-nRNP/Sm, anti-Sm, anti-SSA, anti-Ro-52, anti-SSB, anti-Scl-70, anti-Jo-1, anti-CENP-B, anti-dsDNA, anti-nucleosome, anti-histone, and anti-ribosomal P protein (Rib-P). The detection process included serum incubation, binding, enzyme labeling, color development, and membrane strip scanning, with results automatically interpreted by the EUROLineScan image analysis software. The gray value interpretation criteria were as follows: gray value ≤ 5: negative; 6–10: borderline; > 10: positive. All tests were performed by the Clinical Laboratory Department of our hospital, with positive and negative control groups established, and results confirmed by double review.

*Detection of serum PTX3 and CCL19 levels:* Serum PTX3 and CCL19 levels were quantified by ELISA. Five milliliters of peripheral venous blood was collected from all subjects on the day of outpatient visit or admission. Serum was separated and stored at -80°C to avoid repeated freezing and thawing. The following kits were used: human PTX3 and CCL19 ELISA kits (JL15249-96T, JL13173-96T) purchased from Jonlnbio. Experiments were performed in accordance with the kit instructions, and absorbance values were read at 450 nm using a microplate reader (SpectraMax iD5, Molecular Devices Shanghai Co., Ltd.). A standard curve and blank wells were set for each test, and all samples were tested in duplicate with the average value taken.

### Statistical analysis:

Data analysis was performed using SPSS 27.0 software. Categorical data were expressed as [n (%)] and analyzed by the Chi-square test (*χ^2^* test) or Fisher’s exact test. Continuous data were presented as mean ± standard deviation (*x̄*+*s*), with intergroup comparisons performed by the t-test and multiple group comparisons by one-way analysis of variance (ANOVA). ROC curve analysis was conducted to assess the diagnostic efficacy of ANAs combined with serum PTX3 and CCL19 in SLE, and the 2×2 contingency table method was applied to analyze the diagnostic performance of individual ANAs, PTX3, and CCL19. A *P*-value <0.05 was considered a statistically significant difference.

## RESULTS

There were no significant differences in age, gender, or BMI among the three groups, indicating comparability (all *P*> 0.05). In the experimental group, complement C3, complement C4, platelet count, white blood cell count, and hemoglobin levels were significantly lower than those in both the healthy control and disease control groups, while serum creatinine and blood urea nitrogen levels were significantly higher (all *P*< 0.05). The predominant clinical manifestations of SLE patients were joint involvement, lupus nephritis, and fever, see Table-I.

**Table-I:** Comparison of Clinical Data Among the Three Groups [(*x̄* + *s*)/n (%)].

Indicators	Healthy Control Group (n=60)	Disease Control Group (n=80)	Experimental Group (n=80)	χ^2^/F	P
Age (years)	37.16±9.52	38.24±9.81	37.69±10.22	0.207	0.813
Gender				0.314	0.855
Male	11 (18.33)	14 (17.50)	12 (15.00)		
Female	49 (81.67)	66 (82.50)	68 (85.00)		
BMI (kg/m^2^)	22.41±2.39	22.15±2.64	21.73±2.48	1.319	0.270
Complement C3 (g/L)	1.02±0.09	0.88±0.13*	0.58±0.14*^#^	234.657	<0.001
Complement C4 (g/L)	0.28±0.06	0.21±0.05*	0.09±0.03*^#^	296.278	<0.001
Serum creatinine (μmol/L)	68.61±15.42	73.24±18.76	98.52±26.31*^#^	43.367	<0.001
Blood urea nitrogen (mmol/L)	4.61±1.39	5.28±1.51*	7.64±1.95*^#^	67.914	<0.001
Platelet count (×10^9^/L)	225.37±38.24	213.60±46.82	152.57±41.36*^#^	62.420	<0.001
White blood cell count (×10^9^/L)	6.11±1.42	5.83±1.47	4.29±1.19*^#^	38.632	<0.001
Hemoglobin (g/L)	124.68±19.25	119.27±15.84	102.43±14.27*^#^	36.979	<0.001
Clinical manifestations					
Lupus nephritis	-	-	40 (50.00)	-	-
Neuropsychiatric involvement	-	-	12 (15.00)	-	-
Skin and mucous membrane involvement	-	-	25 (31.25)	-	-
Joint involvement	-	-	58 (72.50)	-	-
Hematological abnormalities	-	-	25 (31.25)	-	-
Fever	-	-	36 (45.00)	-	-

***Note:***

* P < 0.05 compared with the healthy control group;

# P < 0.05 compared with the disease control group.

**Fig.1:**
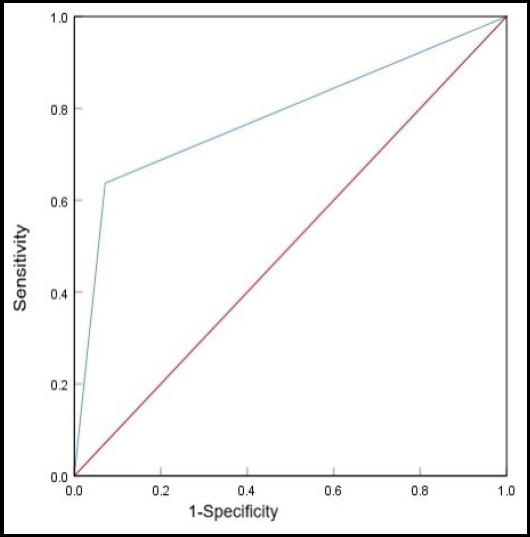
ROC Curve of ANAs for the Diagnosis of SLE.

In the SLE group, the positive rates of anti-nRNP/Sm, anti-Sm, anti-SSA, anti-Ro-52, anti-SSB, anti-dsDNA, anti-nucleosome, anti-histone, and anti-Rib-P were 45.00% (36/80), 30.00% (24/80), 60.00% (48/80), 40.00% (32/80), 35.00% (28/80), 45.00% (36/80), 52.50% (42/80), 27.50% (22/80), and 16.25% (13/80), respectively, all of which were significantly higher than those in the disease control and healthy control groups (all *P* < 0.05).Table-II. The AUC of ANAs for the diagnosis of SLE was 0.783 (*95%CI*: 0.723~0.836), with a sensitivity of 63.75% and a specificity of 92.86%, Fig.1.

**Table-II:** Comparison of ANAs Positivity Rates Among the Three Groups [n (%)]

Autoantibody Types	Healthy Control Group (n=60)	Disease Control Group (n=80)	Experimental Group (n=80)	χ^2^	P
Anti-nRNP/Sm	0 (0.00)	8 (10.00)*	36 (45.00)*^#^	51.250	<0.001
Anti-Sm	0 (0.00)	6 (7.50)*	24 (30.00)*^#^	30.221	<0.001
Anti-SSA	1 (1.67)	16 (20.00)*	48 (60.00)*^#^	61.549	<0.001
Anti-Ro-52	0 (0.00)	15 (18.75)*	32 (40.00)*^#^	33.165	<0.001
Anti-SSB	0 (0.00)	8 (10.00)*	28 (35.00)*^#^	34.408	<0.001
Anti-Scl-70	0 (0.00)	7 (8.75)*	3 (3.75)	4.273	0.118
Anti-Jo-1	0 (0.00)	4 (5.00)	1 (1.25)	2.209	0.331
Anti-CENP-B	0 (0.00)	5 (6.25)*	2 (2.50)	2.625	0.269
Anti-dsDNA	0 (0.00)	10 (12.50)*	36 (45.00)*^#^	47.359	<0.001
Anti-nucleosome	1 (1.67)	15 (18.75)*	42 (52.50)*^#^	49.390	<0.001
Anti-histone	0 (0.00)	8 (10.00)*	22 (27.50)*^#^	23.428	<0.001
Anti-Rib-P	0 (0.00)	4 (5.00)	13 (16.25)*^#^	11.494	0.003

***Note:***

* P<0.05 compared with the healthy control group;

# P<0.05 compared with the disease control group.

**Fig.2:**
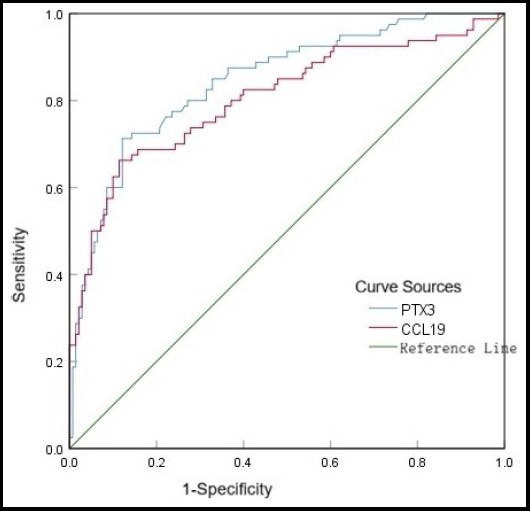
ROC Curves of Serum PTX3 and CCL19 Levels for the Diagnosis of SLE.

Serum PTX3 and CCL19 levels in the experimental group were significantly higher than those in the disease control group and healthy control group (all *P* < 0.05). Table-III. ROC curve analysis revealed that the AUC values of serum PTX3 and CCL19 for SLE diagnosis were 0.845 and 0.805, with sensitivities of 71.25% and 66.25%, specificities of 87.86% and 88.57%, respectively. Table-IV and Fig.2.

**Table-III:** Comparison of Serum PTX3 and CCL19 Levels Among the Three Groups (*x̄* + *s*).

Groups	n	PTX3 (ng/mL)	CCL19 (pg/mL)
Healthy Control Group	60	9.69±2.13	386.33±72.41
Disease Control Group	80	11.83±2.75	437.61±81.96
Experimental Group	80	13.92±3.17	529.78±113.84
*F*	-	40.378	43.863
*P*	-	<0.001	<0.001

**Table-IV:** Diagnostic Efficacy of Serum PTX3 and CCL19 Levels for SLE.

Variables	AUC	Cut-off Value	95%CI	Sensitivity (%)	Specificity (%)	Youden Index
PTX3	0.845	12.88 ng/mL	0.791~0.890	71.25	87.86	0.591
CCL19	0.805	472.28 pg/mL	0.747~0.855	66.25	88.57	0.548

**Fig.3:**
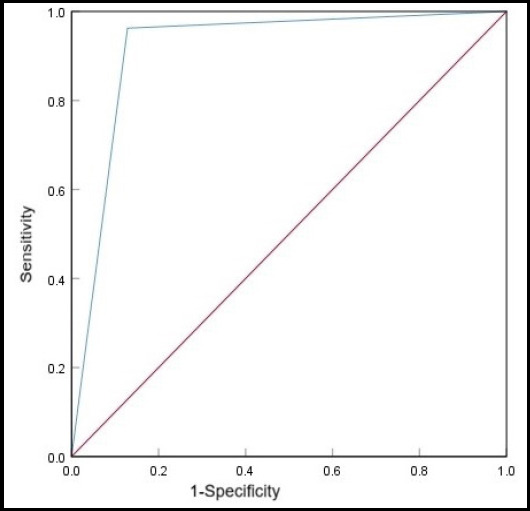
ROC Curve of ANAs Combined with Serum PTX3 and CCL19 Levels for the Diagnosis of SLE.

Compared with the single detection of ANAs, PTX3, or CCL19 alone, the combined detection of these three indicators showed significantly higher sensitivity (96.25%), accuracy (90.45%), and negative predictive value (97.60%) (all *P* < 0.05. Table-V and Fig.3.

**Table-V:** Comparison of Diagnostic Efficacy of ANAs combined with Serum PTX3 and CCL19 Levels for SLE.

Diagnostic Methods	Sensitivity	Specificity	Accuracy	Positive Predictive Value	Negative Predictive Value
ANAs	63.75% (51/80)*	92.86% (130/140)	82.27% (181/220)*	83.61% (51/61)	81.76% (130/159)*
PTX3	71.25% (57/80)*	87.86% (123/140)	81.82% (180/220)*	77.03% (57/74)	84.25% (123/146)*
CCL19	66.25% (53/80)*	88.57% (124/140)	80.45% (177/220)*	76.81% (53/69)	82.12% (124/151)*
Combination of the three	96.25% (77/80)	87.14% (122/140)	90.45% (199/220)	81.05% (77/95)	97.60% (122/125)

***Note:***

* P<0.05 compared with the combination of the three indicators.

## DISCUSSION

In this study, the positive rates of multiple autoantibodies were significantly increased in SLE patients. Among them, anti-SSA (60.00%), anti-nucleosome (52.50%), and anti-dsDNA (45.00%) exhibited relatively high positive rates, suggesting that these autoantibodies play important roles in the immune activation process of SLE. Studies have confirmed that various autoantibodies and inflammatory factors in SLE patients become abnormal in the early stage of the disease, providing a potential breakthrough for early diagnosis,^9^,^10^ which consistent with our study. ANAs reflect B cell-mediated immune abnormalities and are one of the key serological markers for SLE, having been incorporated into multiple SLE classification criteria.^11^ Studies have shown that autoantibodies such as anti-SSA, anti-RNP/Sm, anti-Sm, and anti-dsDNA may emerge in the early stage of SLE. This is particularly meaningful for improving recognition rates when important organs are not yet involved in the initial course of the disease.^12^ Additionally, in this study, the diagnostic sensitivity of ANAs was 63.75%, specificity was 92.86%, and the area under the curve (AUC) was 0.783. While ANAs have high specificity for SLE, their sensitivity is relatively limited. A single antibody may be negative due to differences in patient disease course, disease subtype, or immune response, leading to early missed diagnosis in some patients. Therefore, although ANAs remain valuable for SLE screening and auxiliary diagnosis, relying solely on individual detection has limitations. There is an urgent need to combine them with other biomarkers to improve early recognition rates.^13^-^16^

PTX3 plays a role in autoimmune diseases, and its elevation may indicate disease activity in rheumatoid arthritis, SLE, or other autoimmune disorders^17^. PTX3 can predict target organ damage, regulate bone metabolic immunity, maintain homeostasis, and participate in vascular endothelial remodeling.^17^ Ismail SA et al.^6^ found that serum PTX3 levels were significantly elevated in SLE patients and correlated with disease activity, suggesting that PTX3 can serve as a novel biomarker for monitoring SLE disease status. Di Lorenzo B et al.^18^ reported that blood PTX3 concentrations were significantly increased in patients with rheumatic diseases, playing a role in the pathophysiology of these conditions. The present study demonstrated that serum PTX3 levels in SLE patients were higher than those in the disease control group and healthy control group, indicating that PTX3 may be involved in the immunopathological process of SLE. PTX3 can exacerbate autoimmune responses by promoting phagocyte activation, regulating the complement system, and enhancing inflammatory chemotaxis; its elevation may reflect disease activity or the degree of tissue damage.^6^ ROC curve analysis showed that the AUC of serum PTX3 for SLE diagnosis was 0.845, with a sensitivity of 71.25% and a specificity of 87.86%. This indicates that PTX3 not only serves as an indicator of inflammatory responses but also has high diagnostic potential in autoimmune diseases, especially for patients with early atypical SLE symptoms but elevated inflammatory levels.

CCL19 plays an important role in maintaining lymphoid organ structure, immune cell homing, and adaptive immune responses.^19^ Previous studies have consistently found that serum CCL19 levels are elevated in SLE patients, and CCL19 levels are associated with disease activity and cellular immune dysfunction, potentially serving as a new target for the clinical diagnosis and treatment of SLE.^20^ In this study, serum CCL19 levels in the SLE group were also significantly elevated, and ROC curve analysis showed an AUC of 0.805 for diagnosis, with a sensitivity of 66.25% and a specificity of 88.57%, indicating good discriminative ability. Compared with PTX3, CCL19 has slightly lower sensitivity but similar specificity, suggesting its independent value in identifying SLE. Some studies suggest that the elevation of CCL19 may be related to lymphocyte dysfunction and persistent chronic inflammatory states in SLE patients. CCL19 can promote the migration of T cells and dendritic cells, participate in the formation of an inflammatory microenvironment, and thereby maintain the immune activation state.^7^

Although ANAs, PTX3, and CCL19 each have certain diagnostic capabilities, a single indicator often suffers from insufficient sensitivity or specificity. Further analysis in this study revealed that the combined detection of the three markers yielded significantly higher AUC, sensitivity, and negative predictive value than individual detection. Specifically, the combined diagnosis achieved a sensitivity of 96.25%, accuracy of 90.45%, and negative predictive value of 97.60%, indicating that the combination can effectively compensate for the deficiencies of individual indicators and significantly improve SLE screening efficiency. Combined detection can not only identify patients with positive ANAs but insignificant inflammatory responses but also cover cases with negative ANAs but elevated PTX3 or CCL19, thereby improving the detection rate of atypical or early SLE. Additionally, integrating ANAs with PTX3 and CCL19 reflects the complete pathological process of the disease (from immune abnormalities to inflammatory responses), which is more consistent with the actual pathogenesis of SLE. This combined model provides new insights for establishing a multi-dimensional diagnostic framework and lays a foundation for the clinical promotion of simple and accurate early screening strategies.

### Limitations:

However, this study is a single-center retrospective study with a relatively limited sample size, which may lead to selection bias, and no stratified analysis of patient disease activity was performed. In addition, the specificity of PTX3 and CCL19 needs to be further verified in a larger scope and different disease spectra. Future multi-center, prospective studies can further confirm the stability and promotion of the results of this study.

## CONCLUSIONS

ANAs, serum PTX3, and CCL19 can all serve as potential diagnostic biomarkers for SLE. The combined detection of these three indicators effectively improves the diagnostic efficiency of SLE, which is superior to single-index detection and holds good clinical promotion value. It is particularly suitable for early SLE patients with atypical clinical symptoms and low antibody positive rates, and is expected to reduce missed diagnoses and misdiagnoses.

### Authors’ Contributions:

**QL:** designed the study and conceived the survey, evaluated the results, and are responsible and accountable for the accuracy or integrity of the work.

**JZ** and **ML:** collected epidemiological data. Critical Review.

**DC** and **MZ:** sorted the data, were involved in the fieldwork.

All authors have read and approved the final manuscript.
